# Supplementary material for: The dynamic genetic determinants of increased transcriptional divergence in spermatids
Source: Nat Commun. 2024 Feb 10;15:1272. doi: 10.1038/s41467-024-45133-1 (PMC10858866; doi:10.1038/s41467-024-45133-1)
Supplement: Supplementary file 1 — Supplementary Information [file 41467_2024_45133_MOESM1_ESM.pdf]

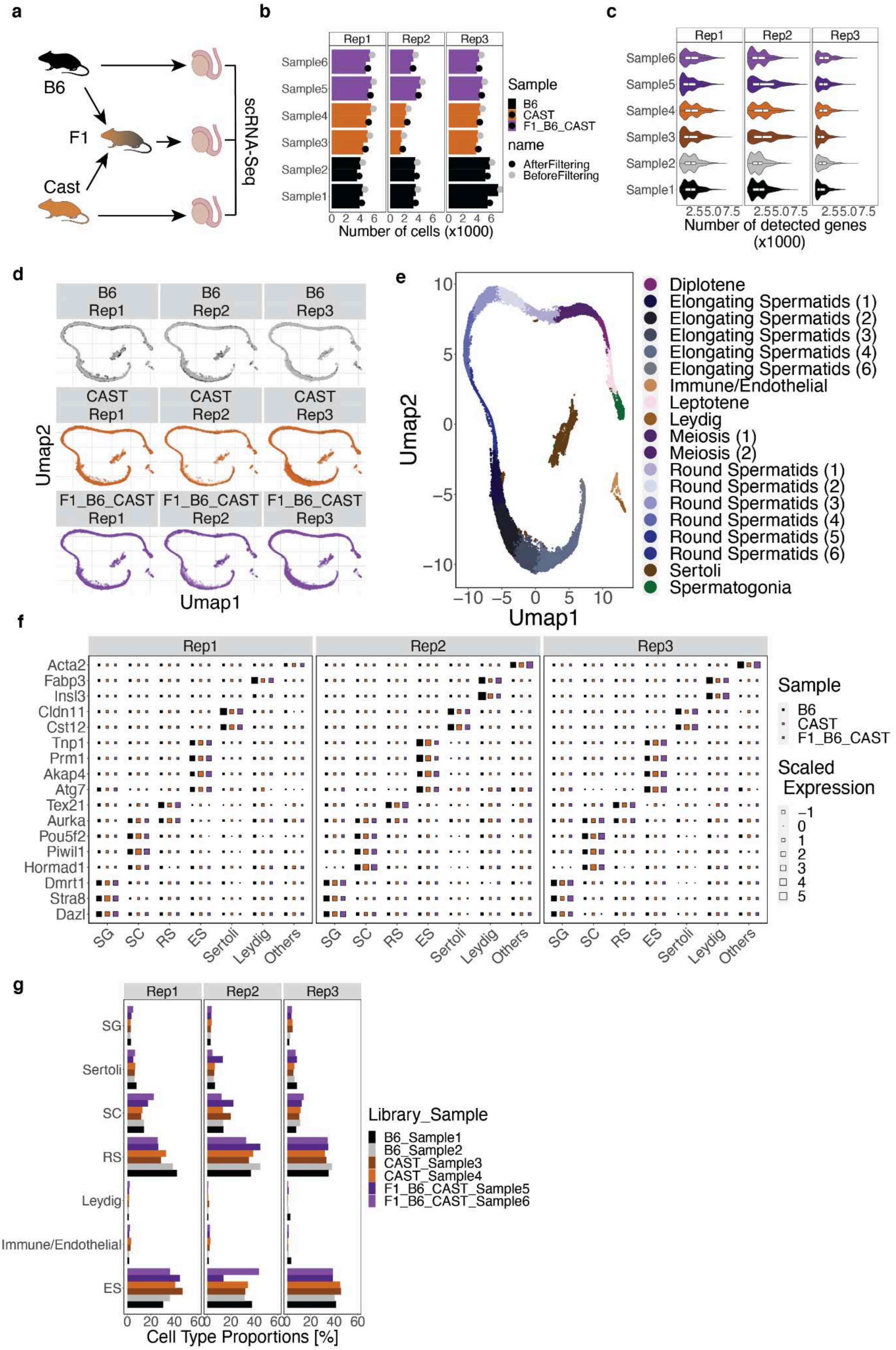

a
B6
F1
Cast
scRNA-Seq
b
Rep1
Rep2
Rep3
Sample6
Sample5
Sample4
Sample3
Sample2
Sample1
Sample
B6
CAST
F1_B6_CAST
name
AfterFiltering
BeforeFiltering
Number of cells (x1000)
c
Number of detected genes (x1000)
d
B6 Rep1
B6 Rep2
B6 Rep3
CAST Rep1
CAST Rep2
CAST Rep3
F1_B6_CAST Rep1
F1_B6_CAST Rep2
F1_B6_CAST Rep3
Umap2
Umap1
e
Diplotene
Elongating Spermatids (1)
Elongating Spermatids (2)
Elongating Spermatids (3)
Elongating Spermatids (4)
Elongating Spermatids (6)
Immune/Endothelial
Leptotene
Leydig
Meiosis (1)
Meiosis (2)
Round Spermatids (1)
Round Spermatids (2)
Round Spermatids (3)
Round Spermatids (4)
Round Spermatids (5)
Round Spermatids (6)
Sertoli
Spermatogonia
f
Acta2
Fabp3
Insl3
Cldn11
Cst12
Tnp1
Prm1
Akap4
Atg7
Tex21
Aurka
Pou5f2
Piwil1
Hormad1
Dmrt1
Stra8
Dazl
SG
SC
RS
ES
Sertoli
Leydig
Others
Scaled Expression
g
Immune/Endothelial
Library_Sample
B6_Sample1
B6_Sample2
CAST_Sample3
CAST_Sample4
F1_B6_CAST_Sample5
F1_B6_CAST_Sample6
Cell Type Proportions [%]

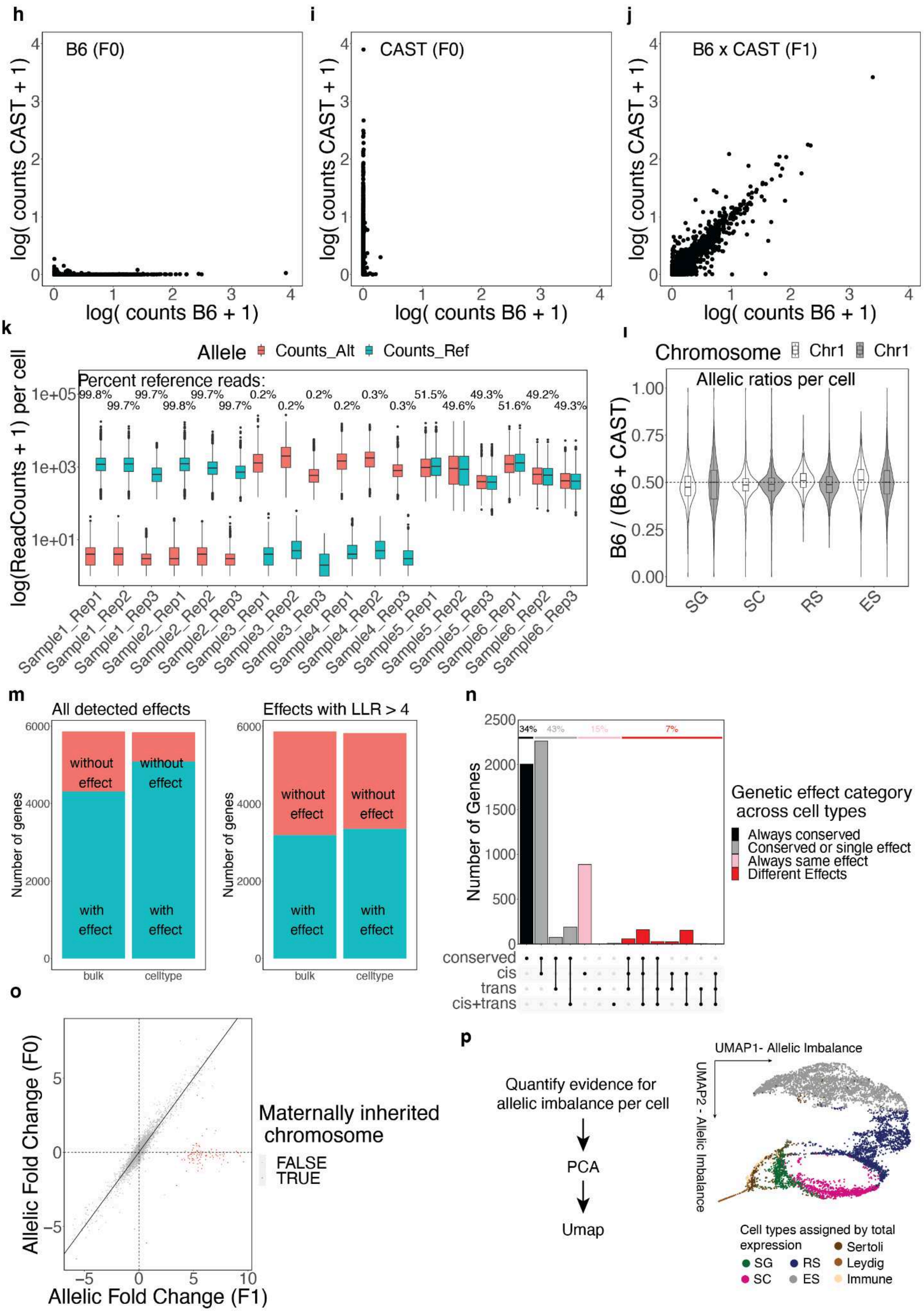
h
B6 (F0)
log(counts CAST + 1)
log(counts B6 + 1)
i
CAST (F0)
log(counts CAST + 1)
log(counts B6 + 1)
j
B6 x CAST (F1)
log(counts CAST + 1)
log(counts B6 + 1)
k
Allele
Counts_Alt
Counts_Ref
Percent reference reads:
99.8% 99.7% 99.7% 99.8% 99.7% 99.7% 0.2% 0.2% 0.2% 0.2% 0.3% 0.3% 51.5% 49.6% 49.3% 51.6% 49.2% 49.3%
log(ReadCounts + 1) per cell
1e+05
1e+03
1e+01
Sample1_Rep1
Sample1_Rep2
Sample1_Rep3
Sample2_Rep1
Sample2_Rep2
Sample2_Rep3
Sample3_Rep1
Sample3_Rep2
Sample3_Rep3
Sample4_Rep1
Sample4_Rep2
Sample4_Rep3
Sample5_Rep1
Sample5_Rep2
Sample5_Rep3
Sample6_Rep1
Sample6_Rep2
Sample6_Rep3
l
Chromosome
Chr1
Chr1
Allelic ratios per cell
B6 / (B6 + CAST)
SG
SC
RS
ES
m
All detected effects
Effects with LLR > 4
Number of genes
without effect
with effect
bulk
celltype
n
34%
43%
15%
7%
Number of Genes
Genetic effect category across cell types
Always conserved
Conserved or single effect
Always same effect
Different Effects
conserved
cis
trans
cis+trans
o
Allelic Fold Change (F0)
Allelic Fold Change (F1)
Maternally inherited chromosome
FALSE
TRUE
p
Quantify evidence for allelic imbalance per cell
PCA
Umap
UMAP1- Allelic Imbalance
UMAP2 - Allelic Imbalance
Cell types assigned by total expression
SG
SC
RS
ES
Sertoli
Leydig
Immune

Supplementary Figure 1: Quality control of scRNA-Seq data and of allele-specific quantifications. (**a**) Schematic overview over the performed experiment. Across plots (a-g and k), Rep1-3 refer to replicate experiments each involving two biological replicate mice per strain. The mouse and testis icons were created with Biorender.com. (**b**) Number of single-cell transcriptomes captured per library before and after filtering out low-complexity cells (detected genes < 100, number of UMIs < 100). (**c**) Violin plots depicting the distribution of UMI counts per cell for each library. (**d**) UMAP-embedding of individual libraries based on MNN-corrected expression values (Methods). Each panel shows two biological replicates for a single strain. (**e**) Joint UMAP-embedding of all cells based on MNN-corrected expression values. Colors show clusters with annotated cell types. (**f**) Dotplot of average log expression values for cell type marker genes (SG - Spermatogonia - *Dazl, Hormad1*; SC - Spermatocytes - *Piwil1, Pou5f2*; RS - Round Spermatids - *Tex21*; ES - Elongating Spermatids - *Prm1*; Sertoli - *Cst12*; Leydig - *Insl3*; Immune/Endothelial - *Acta2*). (**g**) Proportions of major cell types for individual sequencing libraries. (**h-j**) Scatterplots showing the average number of reads mapping to the reference or alternative haplotype for each gene for the parental samples B6 (**h**), CAST (**i**) or the F1 samples (**j**). (**k**) Boxplots of the number of reads assigned to either the reference (B6, maternal) or alternative (CAST, paternal) haplotype per cell for the parental B6 (Sample1/2), parental CAST (Sample3/4) or F1 (Sample5/6) samples. The boxplots show median, 25%- and 75%-quantiles, the whiskers 1.5 inter-quartile ranges. (**l**) Distribution of allelic ratios B6 / (B6 + CAST) across all genes for individual cell types. Haploid spermatids show similar distributions as diploid or meiotic cells. The boxplots show median, 25%- and 75%-quantiles, the whiskers 1.5 inter-quartile ranges. (**m**) Number of genes with assigned regulatory mechanism other than "conserved" (i.e. *cis*, *trans* or *cis+trans*). In the right plot, genes are considered cis if the log likelihood ratio comparing the *cis* to the conserved model exceeds 10, and equivalently for *trans* and *cis+trans*. Barplots show the fraction of genes with an assigned mechanism when performing the analysis jointly across all cells or in individual cell types. In the latter case a gene is considered to have a regulatory mechanism other than conserved if it has this mechanism in any individual cell type. (**n**) Upset-plot showing changes in regulatory category for individual genes between different cell types. By comparing regulatory category assignments in spermatocytes, round spermatids and elongating spermatids, we classify all possible assignments for each gene. Barplots then show the number of genes with different combinations of assignments across cell types, and we highlight genes that are always conserved (black), are conserved or show a single category in some cell types (grey), are always non conserved in the same category (pink) or show multiple non-conserved categories (red). (**o**) Scatterplot comparing allelic fold-changes per gene between F0 and F1. Maternally inherited (mitochondrial and X-linked) genes are highlighted in red and show strong allelic imbalance in the F1. (**p**) UMAP embedding of individual cells as in **Figure 2b**, but based on the evidence for allelic imbalance of individual cells, recapitulates major cell type transitions (**Methods**). Colors represent expression-derived cell types.

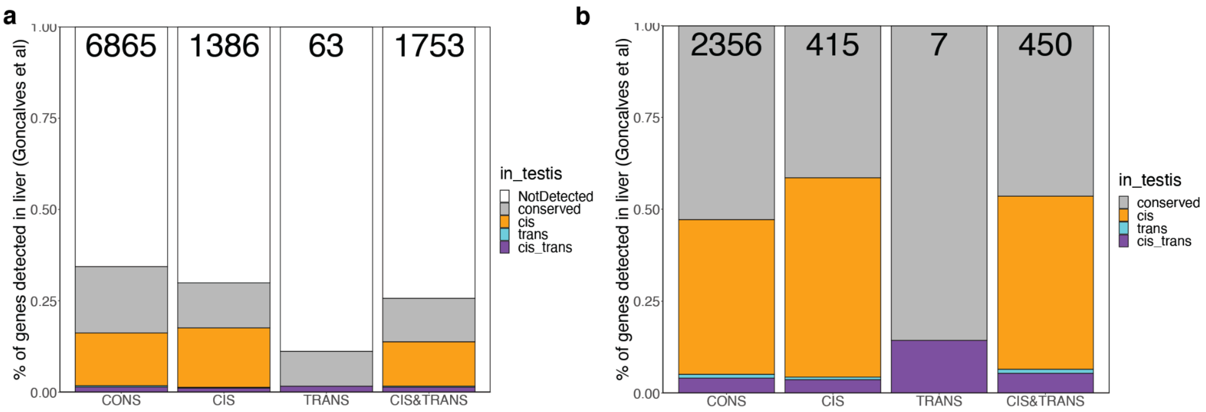

Supplementary Figure 2: Comparison of *cis*- and *trans*-effects with effects in a somatic tissue (Goncalves et al. 2012), demonstrating limited overlap between *cis*- and *trans*-effects identified in different tissues. (a) Stratification of genes with *cis*- and *trans*-effects in liver (x-axis), demonstrating the proportion among each regulatory category which are not detected in testis, or show *cis*- and *trans*-effects in testis (colours). **(b)** As **(a)**, but without genes that are not detected in testis.

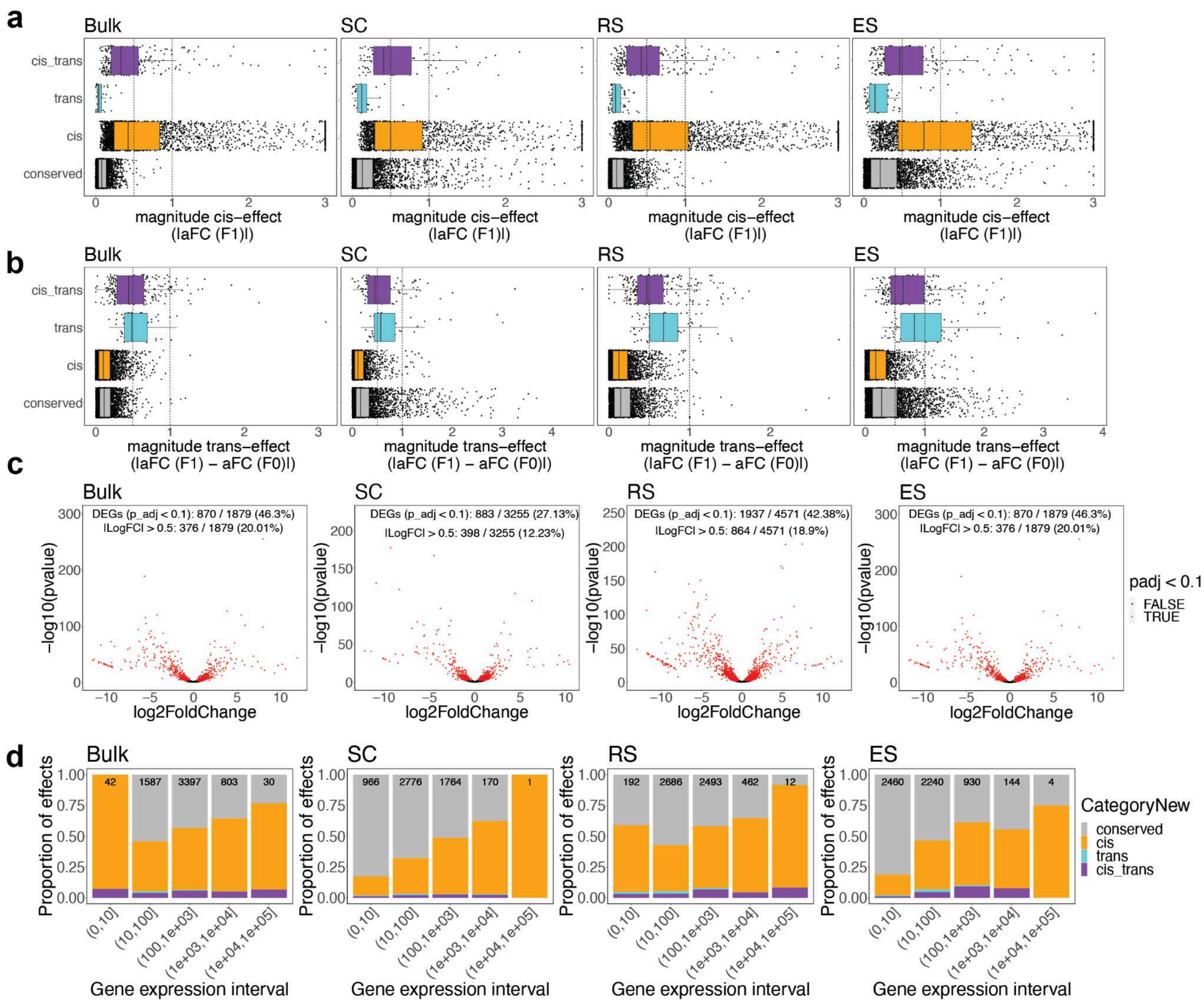

Supplementary Figure 3: Differential expression statistics and effect sizes for cell type-level analysis of *cis*- and *trans*-effects, demonstrating consistency and the presence of substantial allele-specific expression changes. For each analysis, we show the fully combined sample ("Bulk") and individual major cell types (SC: spermatocytes, RS: round spermatids, ES: elongating spermatids) **(a, b)** Effect size magnitude for *cis*- and *trans*-effects, using the the allelic fold changes aFC_F1 = log2 F1_B6 / F1_CAST and aFC_F0 = log F0_B6 / F0_CAST. *Cis*-effect size is calculated as the absolute value of aFC_F1, *trans*-effect magnitudes are calculated as the absolute difference of aFC_F1-aFC_0. **(c)** Classical differential expression analysis between F0 strains using DESeq2. Volcano plots showing differentially expressed genes (DEGs) between B6 and CAST parental strains and a substantial fraction with absolute log allelic fold change > 1. Significant DEGs are shown in red (DESeq2; FDR < 10%). Values over 3 in *cis*-effects are set to 3. The proportion of genes with padj < 0.1 that are classified as non-conserved (see **a**) is 82,2% (bulk), 85,5% (SC), 81,7% (RS) and 77,9% (ES). The boxplots show median, 25%- and 75%-quantiles, the whiskers 1.5 inter-quartile ranges. **(d)** Proportion of genetic effects per regulatory category, stratified by total expression (total number of allele-specific reads) per gene. We find similar proportions of genes assigned to regulatory categories across the spectrum of expression levels, with slightly more effects at higher expression levels.

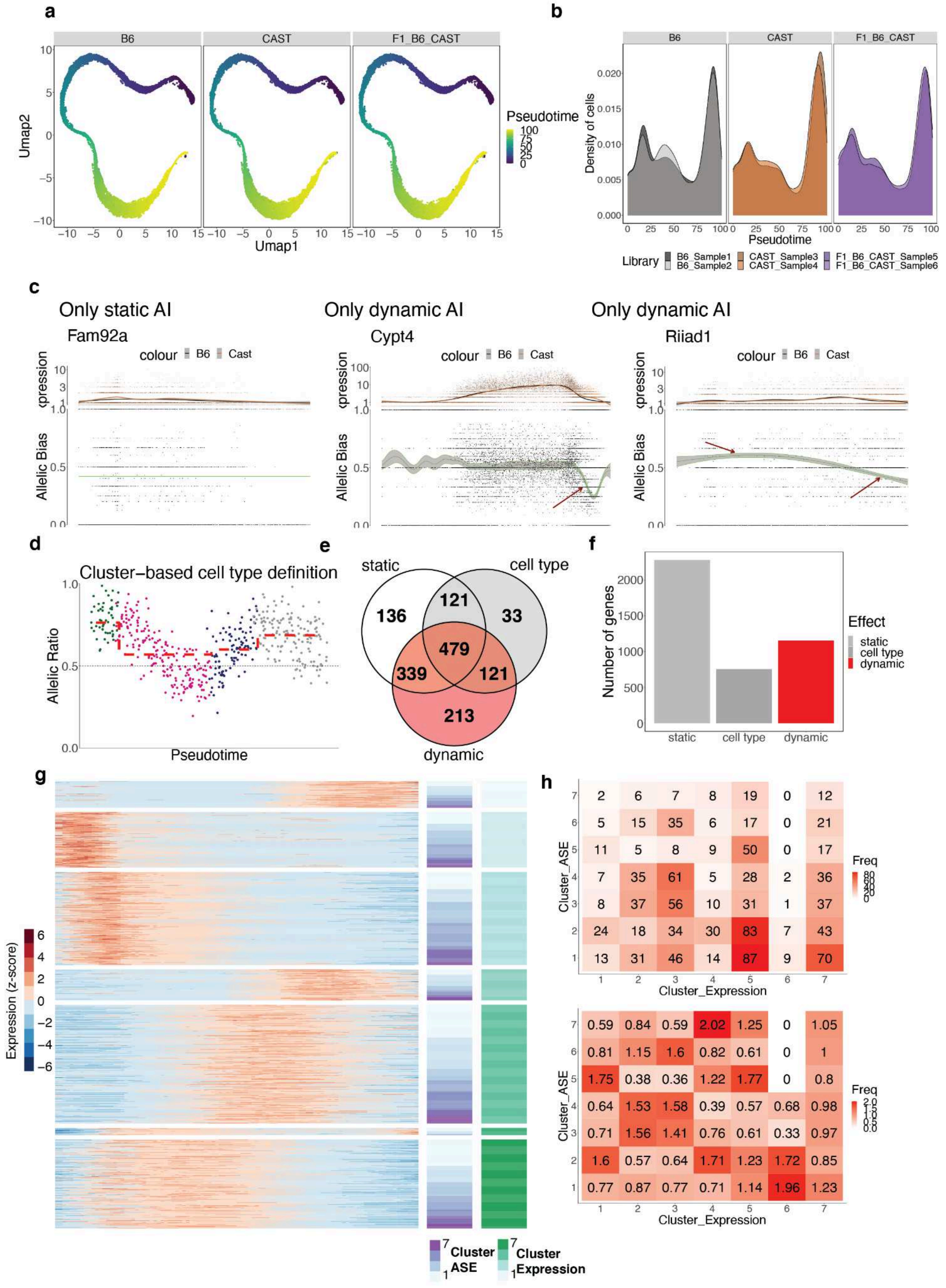

Supplementary Figure 4: Analysis of dynamic allelic imbalance across mouse spermatogenesis. (**a**) UMAP-embedding of mutual nearest neighbor-corrected data across

individual strains, excluding somatic cells. Color shows pseudotemporal ordering of cells based on principal curve fitting. (**b**) Distribution of cells across pseudotime for individual libraries. (**c**) Representations of allelic imbalance trajectories across pseudotime (x-axis) for different genes in F1 mice. In each panel, the top plot shows allelic expression, with each dot representing the log-counts of reads mapping to the given haplotype in a single cell. Smoothed lines denote LOESS-interpolation. In the bottom panel, each dot represents the allelic imbalance B6 / (B6 + CAST) in a single cell, and the green line denotes a gaussian process-interpolated latent allelic trajectory (fitted using scDALI, 2 standard deviations confidence interval of the latent trajectory). Different examples of a gene with only persistent allelic imbalance (*Fam92a*, plot 1), and only dynamic allelic imbalance due to localized effects (*Cypt4*, plot2) or an average allelic balance (*Riiad1*, plot3) are shown. (**d**) Representation of the modeling approach to detect context-specific allelic imbalance using a cluster-based cell type definition. (**e**) Venn-diagram showing the overlap of genes with evidence for allelic imbalance (FDR < 1%) either testing for persistent allelic imbalance (white), using a dynamic allelic imbalance test based on cell type clusters (grey) or testing for dynamic allelic-imbalance based on continuous pseudotime (red). (**f**) As in **e**, showing the total number of genes detected by each test (FDR < 1%). (**g**) Heatmap showing scaled (z-score) average expression values genes across differentiation. Genes are grouped into 7 clusters based on hierarchical clustering. Colored bars on the right represent cluster membership in expression- and ASE-based clustering. (**h**) Confusion matrices showing the overlap of clusters of dynamic genes based on expression (x-axis) or allelic imbalance (y-axis). Top matrix shows the number of overlapping genes per cluster-pair; the right matrix shows the enrichment of genes per cluster based on odds ratios (number of observed genes / number of expected genes).

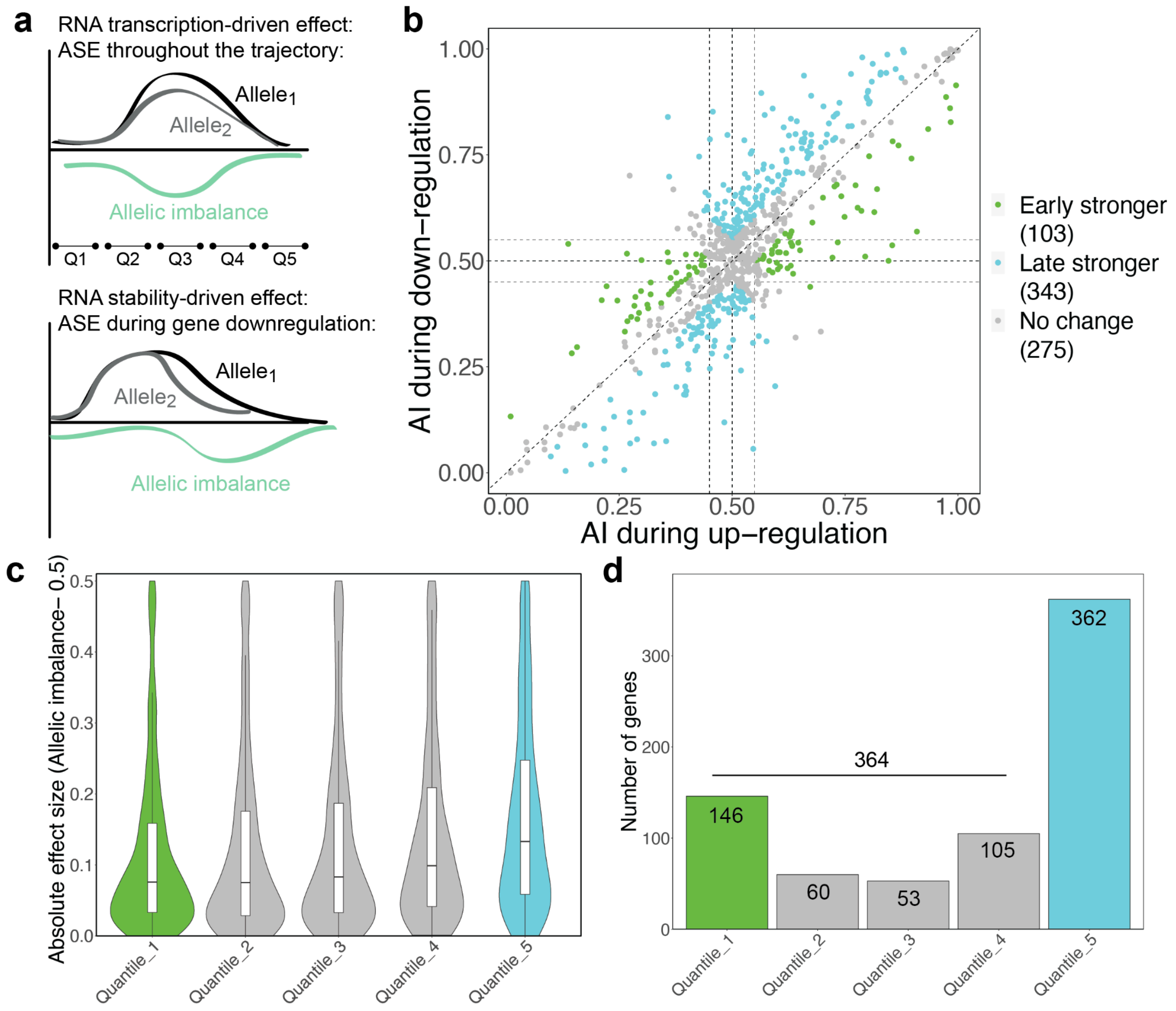

Supplementary Figure 5: Assessment of allelic imbalance in expression during up- and down-regulation of genes during spermatogenesis to differentiate putative transcriptional from RNA-stability associated effects. (a) Cartoon depicting the analysis strategy: The core assumption is that allelic imbalance effects driven by differential RNA stability will be concentrated in the end of the expression trajectory, whereas effects driven by transcriptional regulation can occur anywhere during differentiation. All analysis in this figure is based on a set of 726 genes with dynamic allelic imbalance (scDALI) that show both up- and down-regulation in our dataset **(Fig 2a-d)**. We discretize each expression trajectory into 5 bins (quantiles, Q1-Q5). **(b)** Scatterplot of early versus late allelic expression (x-axis: AI in first quantile of differentiation vs y-axis: AI in last quantile of differentiation). Genes are coded in colour with green denoting genes with stronger early versus late AI and blue denoting genes with stronger late AI versus early (minimal difference of AI > 0.05). **(c)** Violin plots of absolute allelic effect sizes for genes depicted in (b) quantified in all five quantiles (effect size = absolute value of AI - 0.5). The boxplots show median, 25%- and 75%-quantiles, the whiskers 1.5 inter-quartile ranges. **(d)** Quantification of the number of genes for which allelic effects are strongest in the intervals as in **(c)**. While there late allelic effects, which are consistent with allele-specific RNA stability, are frequent (Quantile 5 in **(d)**), there is an equal number of genes that show strongest allelic effects at other stages of differentiation (Quantiles 1-4 in **(d)**), suggesting transcriptional regulation.

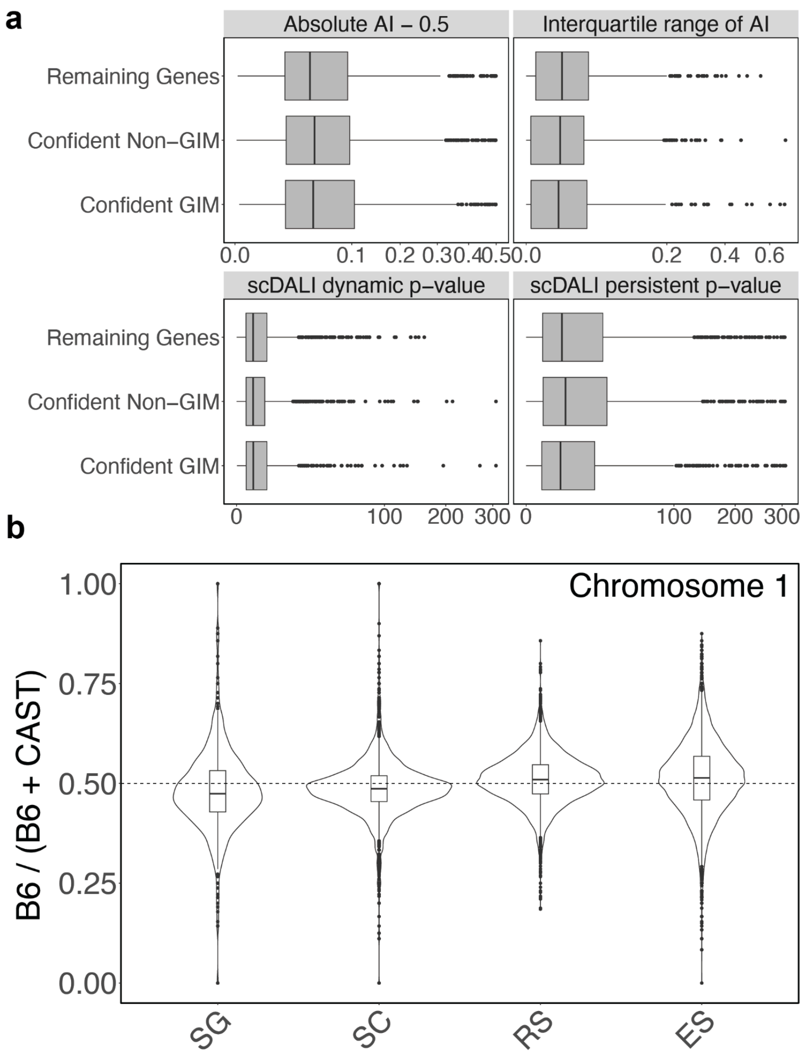

Supplementary Figure 6: *Cis*-effects do not co-localize with transcripts that are unshared through cytoplasmic bridges. (a) We obtained an annotation of genes with shared and unshared transcripts from (Bhutani et al. 2021) and used their classification of confidently genoinformative (transcripts not shared through cytoplasmic bridges), confidently uninformative (fully shared) and remaining genes (unclassified). We then show that both allelic effect sizes and significance estimates by scDALI are equal across groups. Boxplots show (1) absolute a measure of persistent allelic effect size (absolute sample-wide allelic imbalance - 0.5), (2) dynamic allelic effect size ((10%-90%)-interquartile range), (3), distribution of p-values for the dynamic test (dali_pval_linear) across all tested genes and (4) distribution of p-values for the persistent test. None of the differences are significant.

(b) Analysis of chromosome-wide allelic expression ratios across genes on chromosome 1, We observe no differences across germ cell populations of different ploidies (SG: Spermatogonia (2n), SC: Spermatocytes (4n), RS: Round Spermatids (1n), ES: Elongating Spermatids (1n), suggesting that transcripts are predominantly shared across cytoplasmic bridges. All boxplots show median, 25%- and 75%-quantiles, the whiskers 1.5 inter-quartile ranges.

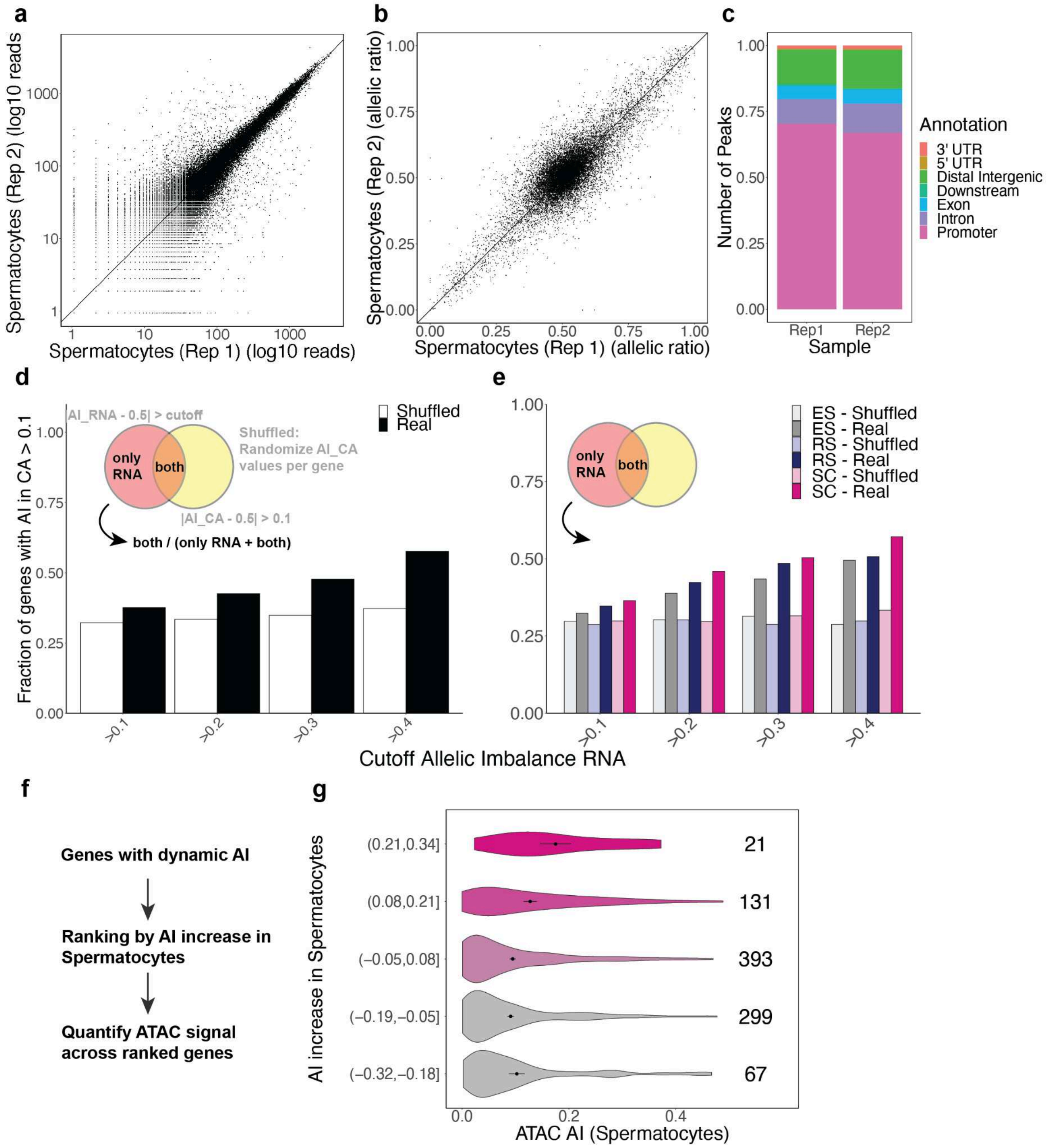

Supplementary Figure 7: Analysis of allele-specific chromatin accessibility (CA) in F1 testis ATAC-Seq data from sorted 4N testicular populations (spermatocytes) in two replicates. (a-c) ATAC-seq quality control, displaying **(a)** scatterplot between per peak read counts between the two biological replicates, **(b)** scatterplot between per peak allelic read count ratios (B6 / (B6 + CAST)) between the two replicates and **(c)** barplot of the genomic distribution of peaks. Collectively, these results demonstrate high reproducibility of the accessibility measurements. (d) Enrichment analysis of co-occurance between allelic imbalance in RNA (AI_RNA) and CA (AI_CA). Shown is the fraction of genes with AI_RNA as defined from sample-wise pseudo-bulk profiles (as used in **Fig 1a-d**) that also harbour at least one proximal (up to 20kb around the transcription start site) accessibility peak with AI_CA (defined as allelic imbalance |AI_CA - 0.5| > 0.1). Genes are stratified by the magnitude of AI_RNA (x-axis) and for the white bars, the assignment of CA peaks to genes

is shuffled, yielding an empirical null distribution. The analysis shows partial coordination between AI in the two omics that increases with the effect size of AI_RNA. **(e)** The analysis in **(d)** is repeated, but using cell type-specific allelic imbalance measurements, showing that AI_CA measured in spermatocytes is more closely corresponding with AI_RNA in spermatocytes, as opposed to other cell types. **(f, g)** Quantification of AI_CA in genes with dynamic AI_RNA. For this analysis, genes are ranked by the difference in AI_RNA in spermatocytes compared to spermatids and the distribution of AI_CA ratios is visualised as a violin plot. Points and error-bars within the violins depict mean and standard deviation. Inserts depict the number of genes in each interval of differential AI_RNA. P-values between the (0.21, 0.34]-Interval and the other intervals are ((0.08,0.21] = 0.063, (-0.05,0.08] = 0.001, (-0.19,-0.05] = 0.006, (-0.32,-0.18] = 0.016) (To-sided Wilcoxon rank sum test, p-values Benjamini-Hochberg adjusted). Error bars represent standard errors.

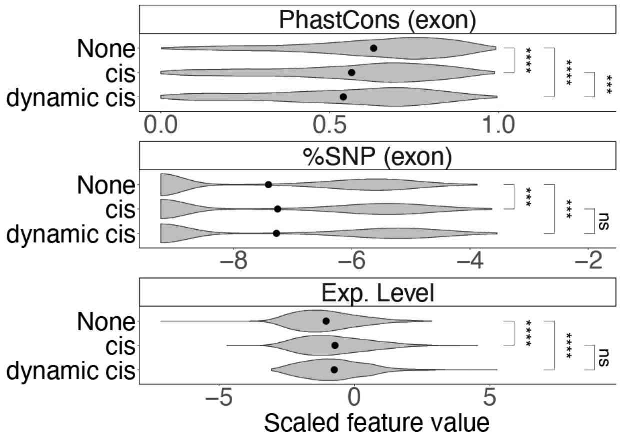

Supplementary Figure 8: Genomic features associated with persistent and dynamic allelic effects. Violin plots showing raw values of genomic features across genes with no, persistent and dynamic allelic effects. *<0.1, **< 0.05, ***< 0.01, ****< 0.001 (Two-sided Wilcoxon's rank sum test). Exact p-values are (from top to bottom): 1.5e-14, 2.2e-16, 0.022, 0.0031, 0.0024, 0.72, 9.9e-12, 8.4e-12, 0.96.

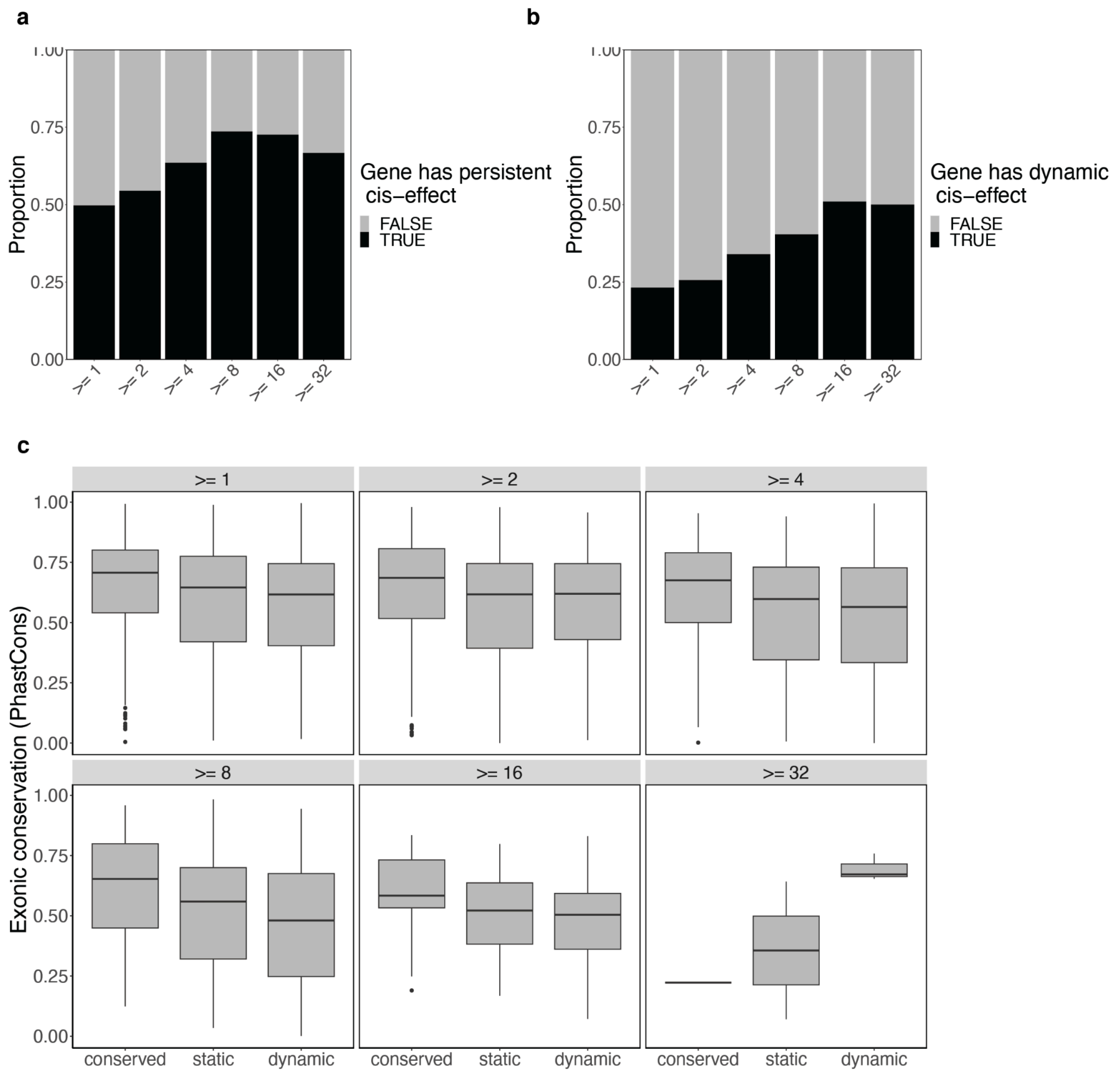

Supplementary Figure 9: Analysis of the number of SNVs per gene based on which allelic imbalance is detected. (a, b) Barplots depicting the fraction of tested genes that show static **(a)** or dynamic **(b)** allelic imbalance. There is a small correlation between the detection of cis-effects and the number of detected SNPs per gene (point-biserial correlation r = 0.15 for persistent, r = 0.14 for dynamic *cis*-effects) **(c)** Boxplots comparing the distribution of exonic PhastCons scores per gene for conserved genes, genes with static and genes with dynamic *cis*-effects, showing similar distribution. This analysis suggests that there is no strong confounding between the number of informative SNVs per gene and the likelihood of cis-effects being detected, and that the decreased sequence conservation of genes with *cis*-effects is observed across different SNV loads. The boxplots show median, 25%- and 75%-quantiles, the whiskers 1.5 inter-quartile ranges.

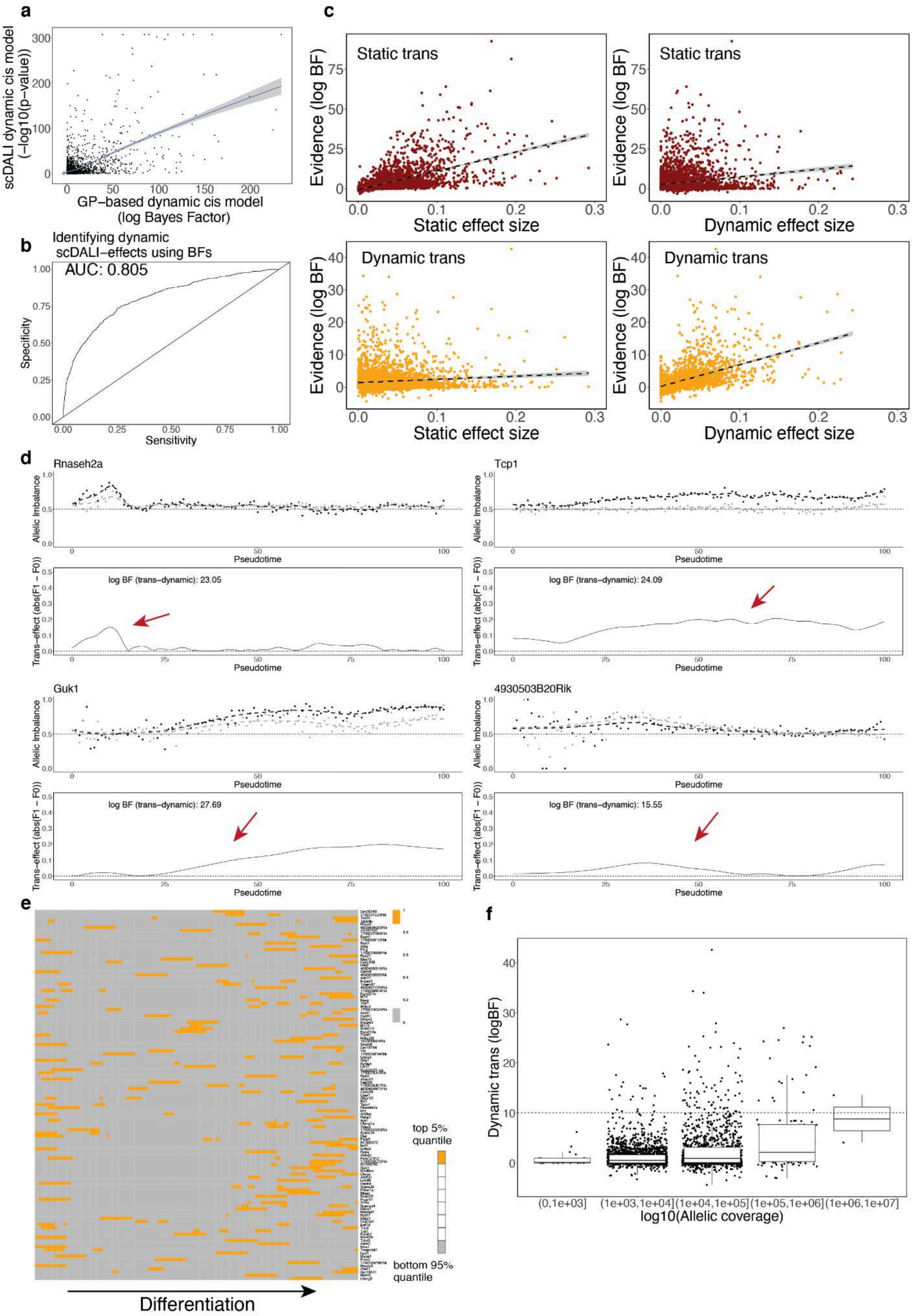

a
scDALI dynamic cis model (−log10(p−value))
GP−based dynamic cis model (log Bayes Factor)
b
Identifying dynamic scDALI−effects using BFs
AUC: 0.805
Specificity
Sensitivity
c
Static trans
Dynamic trans
Evidence (log BF)
Static effect size
Dynamic effect size
d
Rnaseh2a
Tcp1
Guk1
4930503B20Rik
Allelic Imbalance
Trans−effect (abs(F1 − F0))
Pseudotime
log BF (trans−dynamic): 23.05
log BF (trans−dynamic): 24.09
log BF (trans−dynamic): 27.69
log BF (trans−dynamic): 15.55
e
top 5% quantile
bottom 95% quantile
Differentiation
f
Dynamic trans (logBF)
(0,1e+03]
(1e+03,1e+04]
(1e+04,1e+05]
(1e+05,1e+06]
(1e+06,1e+07]
log10(Allelic coverage)

Supplementary Figure 10: Discovery and characterization of dynamic *trans*-effects. (**a**) Scatterplot comparing the detection of dynamic allelic imbalance using the scDALI test (measured by -log10(p-value)) with the Gaussian-process based strategy (measured by log Bayes Factor) outlined in figure 3. (**b**) As in **a**, depicting the detection accuracy of identifying hits from scDALI with the detection from the GP-model (log BF > 10). (**c**) Scatterplots comparing static and dynamic trans-effect sizes (median and qdiff10 of the trans effect F0 - F1 across pseudotime intervals respectively) with evidence based on the GP-model. Evidence is measured as log BF between the static trans vs cis only model (static trans effect) or log BF between the dynamic trans vs static trans model (dynamic trans effect). (**d**) Examples of detected trans-effects. For each gene, the top panel shows average allelic ratios (grey F1, black F0) across cells per pseudotime-bin, with GP-interpolated latent trajectories across pseudotime. The bottom panel shows the absolute difference F1 - F0 between these trajectories. (**e**) Heatmap showing the distribution of trans-effects across spermatogenic differentiation. For each gene (row) the bins with the strongest trans-effects (top 10% quantile) are shown. (**f**) Comparison of log total expression level with allelic resolution against log dynamic BF. Data is shown as box plots across evenly spaced bins of total expression. The boxplots show median, 25%- and 75%-quantiles, the whiskers 1.5 inter-quartile ranges.

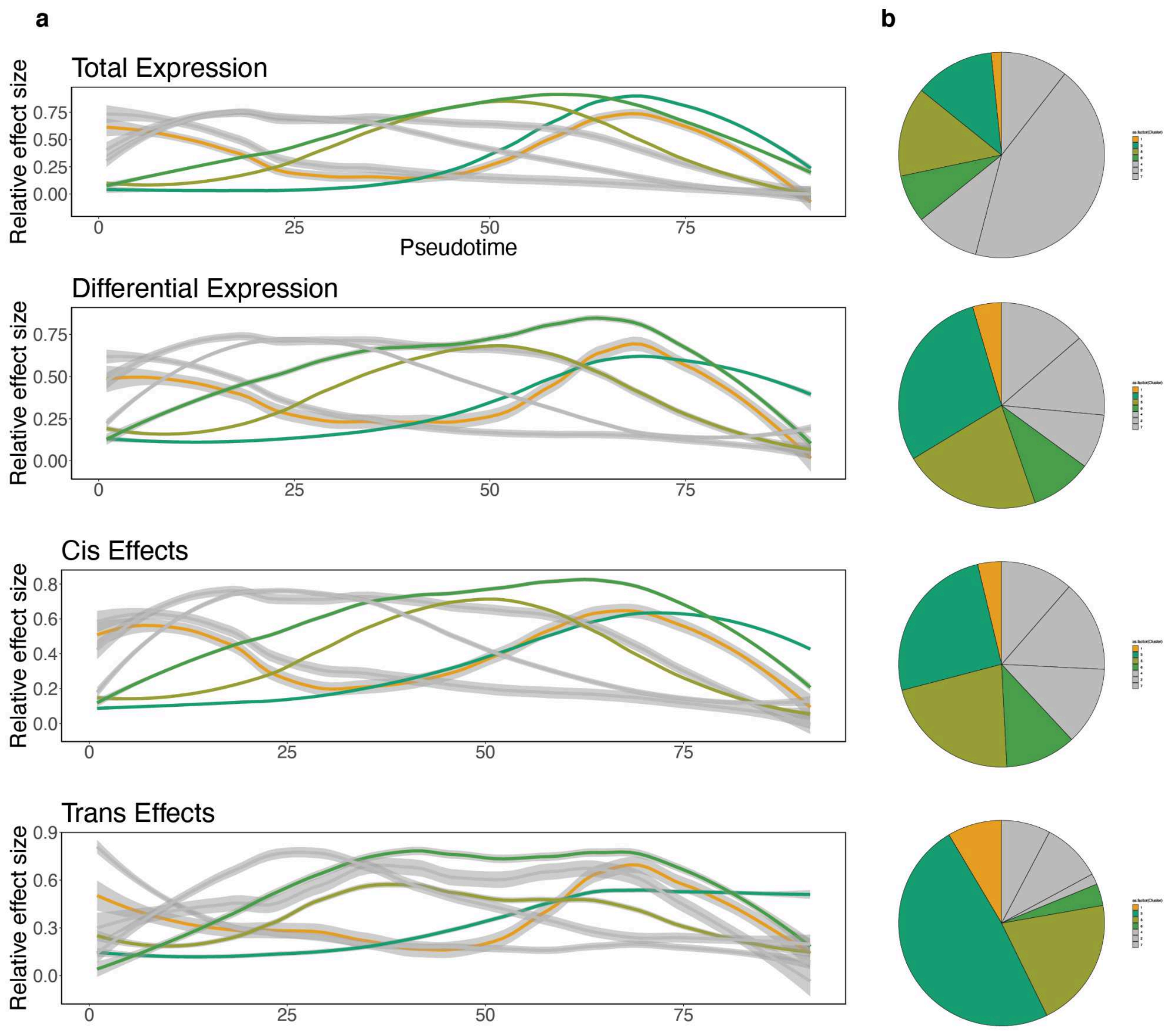

Supplementary Figure 11: Joint clustering of genetic effects and dynamic differential expression. (**a**) Joint clustering of dynamic components in total expression, *cis*-effects, differential expression between strains and *trans*-effects. For each layer, dynamic effect sizes per gene were scaled to a 0-1 range and subjected to hierarchical clustering with a cluster number of 7. Shown are average trajectories for each cluster across pseudotime. Colors show late-peaking clusters. (**b**) Pie charts showing proportions of genes per cluster.

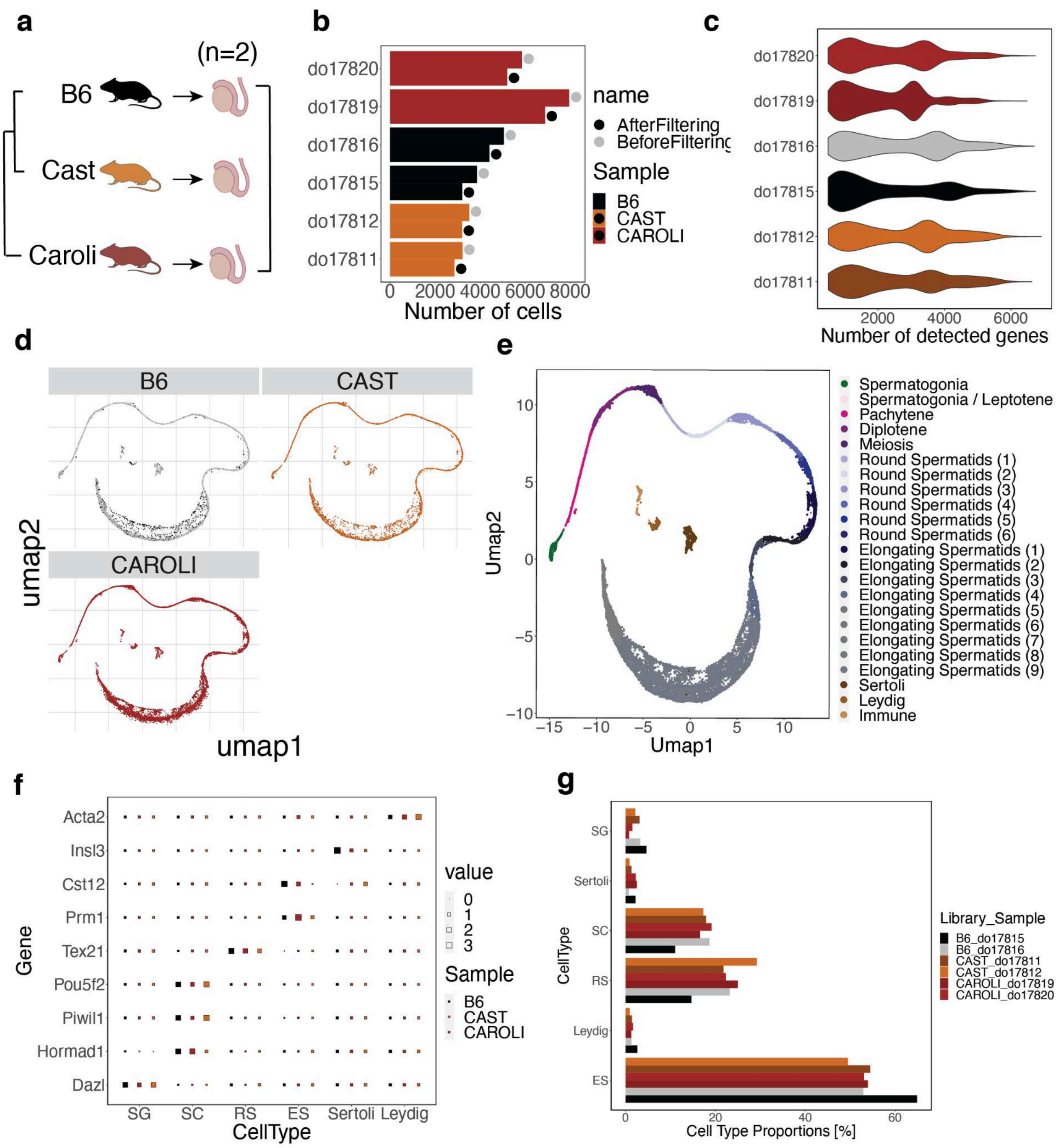
a
(n=2)
B6
Cast
Caroli
b
do17820
do17819
do17816
do17815
do17812
do17811
0
2000 4000 6000 8000
Number of cells
name
AfterFiltering
BeforeFiltering
Sample
B6
CAST
CAROLI
c
Number of detected genes
d
B6
CAST
CAROLI
umap2
umap1
e
Umap2
Umap1
Spermatogonia
Spermatogonia / Leptotene
Pachytene
Diplotene
Meiosis
Round Spermatids (1)
Round Spermatids (2)
Round Spermatids (3)
Round Spermatids (4)
Round Spermatids (5)
Round Spermatids (6)
Elongating Spermatids (1)
Elongating Spermatids (2)
Elongating Spermatids (3)
Elongating Spermatids (4)
Elongating Spermatids (5)
Elongating Spermatids (6)
Elongating Spermatids (7)
Elongating Spermatids (8)
Elongating Spermatids (9)
Sertoli
Leydig
Immune
f
Gene
Acta2
Insl3
Cst12
Prm1
Tex21
Pou5f2
Piwil1
Hormad1
Dazl
SG SC RS ES Sertoli Leydig
CellType
value
0
1
2
3
Sample
B6
CAST
CAROLI
g
CellType
SG
Sertoli
SC
RS
Leydig
ES
0 20 40 60
Cell Type Proportions [%]
Library_Sample
B6_do17815
B6_do17816
CAST_do17811
CAST_do17812
CAROLI_do17819
CAROLI_do17820

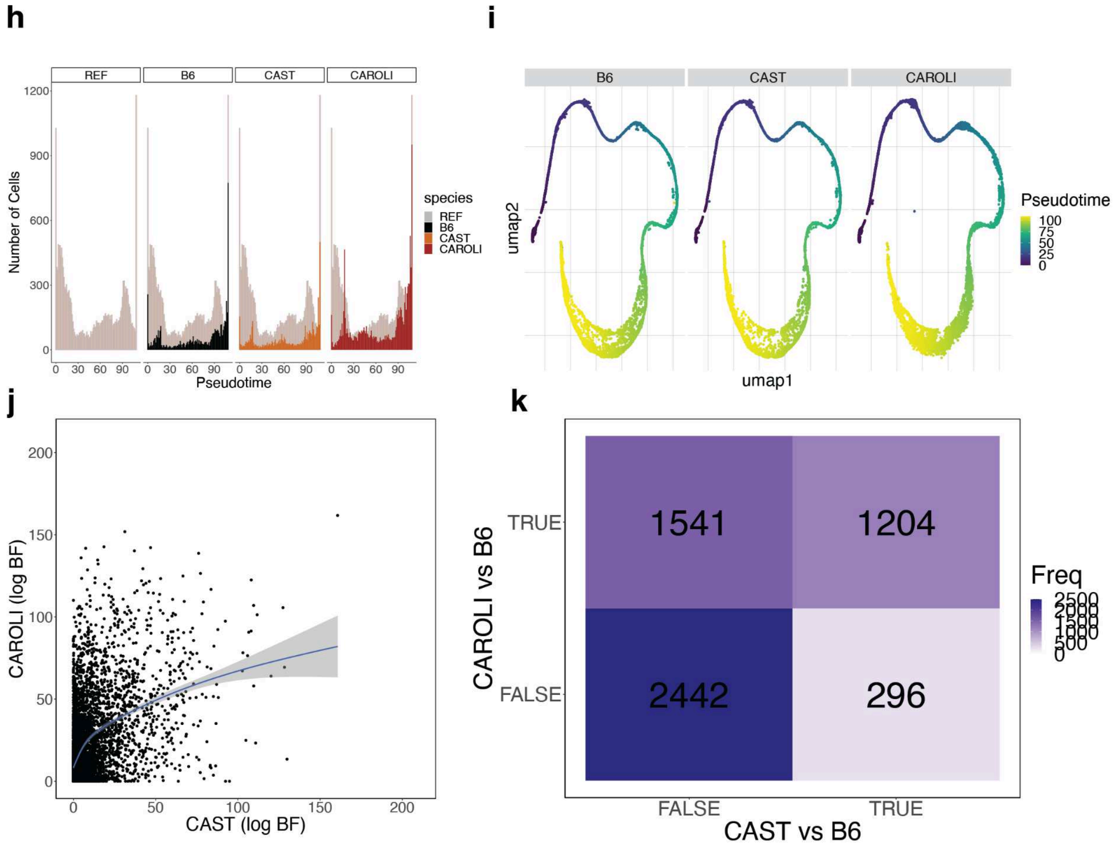

Supplementary Figure 12: Quality control of the independent cross-species dataset and comparison of dynamic differential gene expression. (**a**) Experimental outline for the evolutionary comparison dataset. The mouse and testis icons were created with Biorender.com. n signifies two biological replicate mice. (**b**) Barplots showing the number of cells per library before and after filtering out low-complexity cells (detected genes < 100, number of UMIs < 100). (**c**) Violin plots depicting the distribution of UMI counts per cell for each library. (**d**) UMAP-embedding of individual libraries based on MNN-corrected expression values (**Methods**). Each panel shows two biological replicates for a single strain. (**e**) Joint UMAP-embedding of all cells based on MNN-corrected expression values. Colors show clusters with annotated cell types. (**f**) Dotplot of average log expression values for cell type marker genes (SG - Spermatogonia - *Dazl, Hormad1*; SC - Spermatocytes - *Piwil1, Pou5f2*; RS - Round Spermatids - *Tex21*; ES - Elongating Spermatids - *Prm1*; Sertoli - *Cst12*; Leydig - *Insl3*; Immune/Endothelial - *Acta2*). (**g**) Barplots of cell type distributions for individual libraries, showing the proportion of each major cell type in a given library. (**h**) Histogram of the distribution of pseudotime values based on projecting cells into the reference F1 dataset for a given strain (blue) and the reference F1 dataset (red). (**i**) UMAP-embedding showing pseudotime values across cells. (**j**) Scatterplot showing the evidence for dynamic species specific differential expression across germ cells comparing CAST vs B6 and CAROLI vs B6 comparisons. (**k**) Number of genes considered significant in both comparisons and overlap between comparisons.

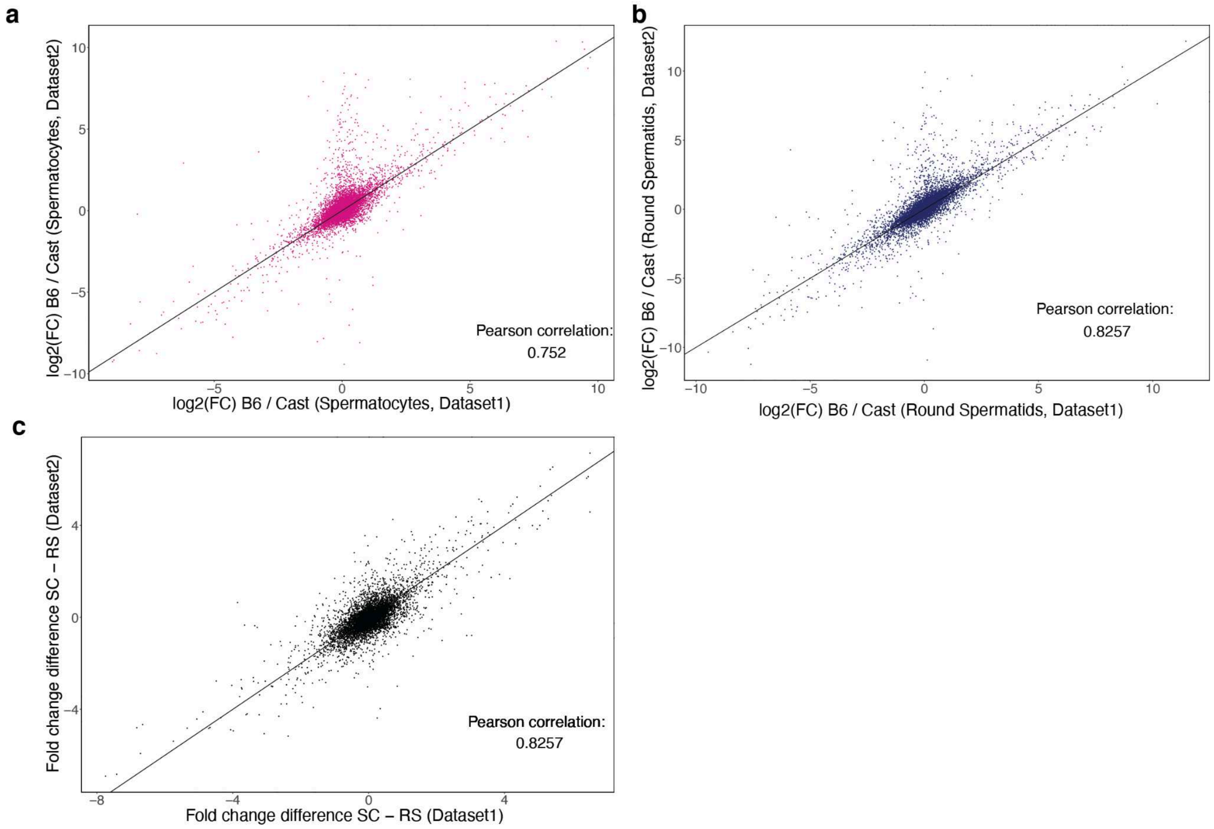

Supplementary Figure 13: Cross-comparison of fold changes between B6 and CAST animals across generated datasets. **(a)** Scatterplot showing expression fold changes between B6 and CAST libraries in spermatid cells between the two datasets. **(b)** As **(a)**, but for round spermatids. **(c)** Scatterplot showing the difference in interspecies fold changes between spermatocytes and spermatids in two datasets, showcasing reproducibility of interspecies and inter-celltype differences.

Supplementary References

Bhutani, Kunal, Katherine Stansifer, Simina Ticau, Lazar Bojic, Alexandra-Chloé Villani, Joanna Slisz, Claudia M. Cremers, et al. 2021. “Widespread Haploid-Biased Gene Expression Enables Sperm-Level Natural Selection.” *Science* 371 (6533). https://doi.org/10.1126/science.abb1723.

Goncalves, Angela, Sarah Leigh-Brown, David Thybert, Klara Stefflova, Ernest Turro, Paul Flicek, Alvis Brazma, Duncan T. Odom, and John C. Marioni. 2012. “Extensive Compensatory Cis-Trans Regulation in the Evolution of Mouse Gene Expression.” *Genome Research* 22 (12): 2376–84.

Supplementary Methods

Jasper Panten

January 8, 2024

1 Categorization of effects into *cis* and *trans* in pseudo-bulk populations

Based on F0 and F1 bulk or pseudo-bulk measurements, genes can be categorized as having *cis*- and *trans*-effects based on statistical models as presented previously in Goncalves et al. (2012); here, we follow the same strategy. Briefly, we generate pseudo-bulk libraries for each allele by summing reads across all cells within a library and a cell type. For the F0 samples, we further divide the observed counts by a scaling factor proportional to the total number of reads, to account for potential differences in library size. Suppose for a given gene we have x_i, y_j normalized read counts in F0 species 1 and 2 (B6 and CAST respectively) for replicates i and j, as well as k_l reads aligning to the reference and n_l reads aligning to the alternative allele in F1 sample l. We assume the following distributions for the counts:

$$\begin{aligned} x_i &\sim \text{Poi}(\lambda_x), \lambda_x \sim \text{Ga}(r, p_x/(1-p_x)) \\ y_i &\sim \text{Poi}(\lambda_y), \lambda_y \sim \text{Ga}(r, p_y/(1-p_y)) \\ k_l &\sim \text{Bin}(n_l, p_l), p_l \sim \text{Beta}(\pi/\theta, (1-\pi)/\theta) \end{aligned} \tag{1}$$

which yield negative and beta-binomial marginal distributions for x_i, y_i and k_i respectively, both of which model unobserved variation encoded by r for the Gamma- and θ for the Beta-distribution. We then consider that genes can be conserved across species, be regulated in *cis*, in *trans* or in both *cis* and *trans*. Each of these categories leads to restrictions on the parameters in the above model in the following way:

conserved: No difference in expression levels between alleles in F0 or F1,
$p_x = p_y$ and $\pi = 0.5$

cis: Different expression levels between alleles, with the same difference in F0 and F1,
$p_x \neq p_y$ and $\pi = p_x/(1-p_x)/(p_x/(1-p_x) + p_y/(1-p_y))$

trans: No difference in expression levels between alleles in F1 but a difference in F0,
$p_x \neq p_y$ and $\pi = 0.5$

cis + trans: No difference in expression levels between alleles in F0 or F1,
$p_x = p_y$ and $\pi \neq 0.5$

We fit the parameters for each model for each gene across replicates using maximum likelihood estimation and use Akaikes information criterion to choose the best model. For the analysis in Figure S1m, we increase the assignment stringency by requiring a threshold in the log-likelihood ratio >10 between a given model and the conserved null-model.

2 Detecting static and dynamic *cis*- and *trans*-effects using multi-output Gaussian proccess models

As in the first section, consider x_i, y_j and k_l, n_l as the read numbers from both F0 strains and the F1 for a given replicate (which could be a single cell, aggregated across cell types or entire libraries). In scDALI, cell type-dependencies of allelic imbalance across single cells are encoded using principles from Gaussian process (GP) regression (Rasmussen and Williams (2006)). In particular, scDALI assumes that for a given gene

$$k_l \sim \text{Bin}(n_l, p_l) \tag{2}$$
$$p_l \sim \text{Beta}(r_l/\theta, (1-r_l)/\theta) \tag{3}$$
$$\text{logit}(\mathbf{r}) = \mathbf{u} \tag{4}$$
$$\mathbf{u} \sim \mathcal{N}(\alpha \mathbf{1}, \sigma^2 \mathbf{K}) \tag{5}$$

where $\mathbf{r} = (r_1, r_2, ..., r_n)$ is the (unobserved) vector of allelic rates, $\mathbf{K}$ is a kernel matrix representing continuous or discrete cell state labels, α represents a null allelic rate per cell and θ is the over-dispersion parameter of the Beta-distribution (Heinen et al. (2022)).
Here, we extend this strategy to jointly model cell type-specific variation in allelic balance between F0 and F1 mice across pseudo-temporal ordering by using a multi-output GP model, also known as the intrinsic model of co-regionalization (Alvarez et al. (2011)). To define a common coordinate system between F0 and F1 samples, we define evenly spaced timepoints $t_0 < t_1 < ... < t_{100}$ across pseudo-time and compute average observed allelic rates in each interval $[t_i, t_{i+1}]$

$$r_{F0}^{t_i} = \frac{\sum x_c}{\sum x_c + \sum y_c}, r_{F1}^{t_i} = \frac{\sum k_c}{\sum n_c}, \tag{6}$$

where the index c considers all cells in the interval t_i. We then define for $\mathbf{r}_{F0} = (r_{F0}^1, r_{F0}^2, ..., r_{F0}^n)$ and $\mathbf{r}_{F1} = (r_{F1}^1, r_{F1}^2, ..., r_{F1}^n)$

$$\text{logit}(\mathbf{r}_{F0}) = \mathbf{u}_{F0} \tag{7}$$
$$\text{logit}(\mathbf{r}_{F1}) = \mathbf{u}_{F1} \tag{8}$$

and assume

$$\begin{pmatrix} \mathbf{u}_{F_0} \\ \mathbf{u}_{F_1} \end{pmatrix} \sim \mathcal{N}\left(\begin{pmatrix} \boldsymbol{\mu}_{F_0} \\ \boldsymbol{\mu}_{F_1} \end{pmatrix}, \begin{pmatrix} \mathbf{K}_{F_0,F_0} & \mathbf{K}_{F_1,F_0} \\ \mathbf{K}_{F_0,F_1} & \mathbf{K}_{F_1,F_1} \end{pmatrix} + \begin{pmatrix} \sigma_1 \mathbf{I} & \mathbf{0} \\ \mathbf{0} & \sigma_2 \mathbf{I} \end{pmatrix} \right) \tag{9}$$

Here, $\boldsymbol{\mu}_{F_0}$ and $\boldsymbol{\mu}_{F_1}$ represent constant mean functions that model shifts of the allelic imbalance trajectory from 0.5, $\mathbf{K}_{F_0,F_0}$ and $\mathbf{K}_{F_1,F_1}$ represent the covariance functions of the F0 and F1 trajectories respectively and $\mathbf{K}_{F_1,F_0}$, $\mathbf{K}_{F_0,F_1}$ are cross-covariance functions modelling correlated or uncorrelated behaviour of the F0 and F1 functions. The normal approximation to the Beta-binomial likelihood in (2) and (3) is justified for high total counts $\sum x_c + \sum y_c$ and $\sum n_c$. We therefore only consider bins with at least 100 aggregated reads across F0 and F1. We further assume a single kernel function $\mathbf{K}$ with joint hyper-parameters across F0 and F1 (but potentially different realizations), allowing us to write

$$\begin{aligned}
\mathcal{N}\left(\begin{pmatrix} \boldsymbol{\mu}_{F_0} \\ \boldsymbol{\mu}_{F_1} \end{pmatrix}, \begin{pmatrix} \mathbf{K}_{F_0,F_0} & \mathbf{K}_{F_1,F_0} \\ \mathbf{K}_{F_0,F_1} & \mathbf{K}_{F_1,F_1} \end{pmatrix} + \begin{pmatrix} \sigma_1 \mathbf{I} & \mathbf{0} \\ \mathbf{0} & \sigma_2 \mathbf{I} \end{pmatrix} \right) &= \mathcal{N}\left(\begin{pmatrix} \boldsymbol{\mu}_{F_0} \\ \boldsymbol{\mu}_{F_1} \end{pmatrix}, \begin{pmatrix} \sigma_{F_0,F_0}\mathbf{K} & \sigma_{F_1,F_0}\mathbf{K} \\ \sigma_{F_0,F_1}\mathbf{K} & \sigma_{F_1,F_1}\mathbf{K} \end{pmatrix} + \begin{pmatrix} \sigma_1 \mathbf{I} & \mathbf{0} \\ \mathbf{0} & \sigma_2 \mathbf{I} \end{pmatrix} \right) \\
&= \mathcal{N}\left(\begin{pmatrix} \boldsymbol{\mu}_{F_0} \\ \boldsymbol{\mu}_{F_1} \end{pmatrix}, \begin{pmatrix} \sigma_{F_0,F_0} & \sigma_{F_1,F_0} \\ \sigma_{F_0,F_1} & \sigma_{F_1,F_1} \end{pmatrix} \otimes \mathbf{K} + \begin{pmatrix} \sigma_1 & 0 \\ 0 & \sigma_2 \end{pmatrix} \otimes \mathbf{I} \right) \\
&= \mathcal{N}\left(\begin{pmatrix} \boldsymbol{\mu}_{F_0} \\ \boldsymbol{\mu}_{F_1} \end{pmatrix}, \mathbf{C} \otimes \mathbf{K} + \begin{pmatrix} \sigma_1 & 0 \\ 0 & \sigma_2 \end{pmatrix} \otimes \mathbf{I} \right)
\end{aligned} \tag{10}$$

where $\otimes$ is the kronecker product and σ_1 and σ_2 encode residual variation not explained by the cell state. Based on this general GP model, we can now derive specific models for static and dynamic trans effects:

no or only cis effects (1): No difference in allelic trajectory between F1 and F0:

$$\boldsymbol{\mu}_{F_0} = \boldsymbol{\mu}_{F_1}, \mathbf{C} = \begin{pmatrix} 1 & 1 \\ 1 & 1 \end{pmatrix}$$

only static trans-effect (2): Only constant shift between F0 and F1:

$$\boldsymbol{\mu}_{F_0} \neq \boldsymbol{\mu}_{F_1}, \mathbf{C} = \begin{pmatrix} 1 & 1 \\ 1 & 1 \end{pmatrix}$$

static + dynamic trans-effect (3): Variable allelic trajectories:

$$\boldsymbol{\mu}_{F_0} \neq \boldsymbol{\mu}_{F_1}, \mathbf{C} = \begin{pmatrix} \sigma_{F_0,F_0} & \sigma_{F_1,F_0} \\ \sigma_{F_0,F_1} & \sigma_{F_1,F_1} \end{pmatrix}$$

We fit all models using variational Gaussian processes (VGP model in the package gpflow) and use the ELBO (evidence lower bound) as an approximation to the marginal likelihood of the data under each model. Based on this, we define Bayes Factors as the ratio of these marginal likelihoods between two models. We define evidence for static trans-effects as the Bayes factor for model **(2)** against model **(1)** and dynamic trans-effects as evidence for model **(3)** against model **(1)**. Moreover, this framework allows to detect dynamic cis effects by substituting $\mathbf{K}$ with a constant kernel.

References

M. A. Alvarez, L. Rosasco, and N. D. Lawrence, "Kernels for Vector-Valued functions: a review," Jun. 2011.

A. Goncalves, S. Leigh-Brown, D. Thybert, K. Stefflova, E. Turro, P. Flicek, A. Brazma, D. T. Odom, and J. C. Marioni, "Extensive compensatory cis-trans regulation in the evolution of mouse gene expression," *Genome Res.*, vol. 22, no. 12, pp. 2376–2384, Dec. 2012.

T. Heinen, S. Secchia, J. P. Reddington, B. Zhao, E. E. M. Furlong, and O. Stegle, "scDALI: modeling allelic heterogeneity in single cells reveals context-specific genetic regulation," *Genome Biol.*, vol. 23, no. 1, p. 8, Jan. 2022.

C. E. Rasmussen and C. K. I. Williams, *Gaussian processes for machine learning.*, ser. Adaptive computation and machine learning. MIT Press, 2006.
