# Supplementary material for: The dynamic genetic determinants of increased transcriptional divergence in spermatids
Source: Nat Commun. 2024 Feb 10;15:1272. doi: 10.1038/s41467-024-45133-1 (PMC10858866; doi:10.1038/s41467-024-45133-1)
Supplement: Supplementary file 3 — Description of Additional Supplementary Files [file 41467_2024_45133_MOESM3_ESM.pdf]

Description of Additional Supplementary Files

File Name: Supplementary Data 1
Description: Table of all individual mice used in this study for single-cell RNA-Sequencing.
