# Supplementary material for: The dynamic genetic determinants of increased transcriptional divergence in spermatids
Source: Nat Commun. 2024 Feb 10;15:1272. doi: 10.1038/s41467-024-45133-1 (PMC10858866; doi:10.1038/s41467-024-45133-1)
Supplement: Supplementary file 5 — Reporting Summary [file 41467_2024_45133_MOESM5_ESM.pdf]

nature portfolio

Corresponding author(s): Prof. Duncan Odom, Prof. Oliver Stegle

Last updated by author(s): Nov 22, 2023

Reporting Summary

Nature Portfolio wishes to improve the reproducibility of the work that we publish. This form provides structure for consistency and transparency in reporting. For further information on Nature Portfolio policies, see our Editorial Policies and the Editorial Policy Checklist.

Statistics

For all statistical analyses, confirm that the following items are present in the figure legend, table legend, main text, or Methods section.

n/a	Confirmed	
☐	☒	The exact sample size (*n*) for each experimental group/condition, given as a discrete number and unit of measurement
☐	☒	A statement on whether measurements were taken from distinct samples or whether the same sample was measured repeatedly
☐	☒	The statistical test(s) used AND whether they are one- or two-sided<br>*Only common tests should be described solely by name; describe more complex techniques in the Methods section.*
☐	☒	A description of all covariates tested
☐	☒	A description of any assumptions or corrections, such as tests of normality and adjustment for multiple comparisons
☐	☒	A full description of the statistical parameters including central tendency (e.g. means) or other basic estimates (e.g. regression coefficient) AND variation (e.g. standard deviation) or associated estimates of uncertainty (e.g. confidence intervals)
☐	☒	For null hypothesis testing, the test statistic (e.g. *F*, *t*, *r*) with confidence intervals, effect sizes, degrees of freedom and *P* value noted<br>*Give P values as exact values whenever suitable.*
☒	☐	For Bayesian analysis, information on the choice of priors and Markov chain Monte Carlo settings
☒	☐	For hierarchical and complex designs, identification of the appropriate level for tests and full reporting of outcomes
☐	☒	Estimates of effect sizes (e.g. Cohen's *d*, Pearson's *r*), indicating how they were calculated

Our web collection on statistics for biologists contains articles on many of the points above.

Software and code

Policy information about availability of computer code

Data collection	No software was used for data collection.
Data analysis	Published software: Processing tools for sequencing data (CellRanger, v3.1) as well as analysis tools written in R (scran v1.20.1, scater v1.20.1, Rtsne, v0.15, umap, v0.2.7.0, igraph v1.2.10, princurve v2.1.6, stats v4.4.2, DESeq2 v1.32.0, VGAM v1.1.5, GenomicRanges, v1.44.0, ChIPseeker v1.28.3,) and python (GPflow (v2.1.4)) and command line tools (trimmomatic v0.38, bowtie2 v2.3.5.1, macs2 v2.1.2.1, WASP v0.3.4). Custom analysis scripts and code to reproduce all figures is available at https://github.com/PMBio/ase_spermatogenesis.

For manuscripts utilizing custom algorithms or software that are central to the research but not yet described in published literature, software must be made available to editors and reviewers. We strongly encourage code deposition in a community repository (e.g. GitHub). See the Nature Portfolio guidelines for submitting code & software for further information.

Data

Policy information about availability of data

All manuscripts must include a data availability statement. This statement should provide the following information, where applicable:

- Accession codes, unique identifiers, or web links for publicly available datasets
- A description of any restrictions on data availability
- For clinical datasets or third party data, please ensure that the statement adheres to our policy

All newly generated sequencing data has been deposited in ArrayExpress under the accession number E-MTAB-11602 [https://www.ebi.ac.uk/biostudies/

arrayexpress/studies/E-MTAB-11602]. The B6 samples of the cross-species comparison are deposited in ArrayExpress under the accession number E-MTAB-6934 [https://www.ebi.ac.uk/biostudies/arrayexpress/studies/E-MTAB-6934]. The spermatocyte ATAC-Seq has been deposited under the accession number E-MTAB-12685 [https://www.ebi.ac.uk/biostudies/arrayexpress/studies/E-MTAB-12685]. All other relevant data supporting the key findings of this study are available
All newly generated sequencing data has been deposited in ArrayExpress under the accession number E-MTAB-11602 [https://www.ebi.ac.uk/biostudies/
arrayexpress/studies/E-MTAB-11602]. The B6 samples of the cross-species comparison are deposited in ArrayExpress under the accession number E-MTAB-6934 [https://www.ebi.ac.uk/biostudies/arrayexpress/studies/E-MTAB-6934]. The spermatocyte ATAC-Seq has been deposited under the accession number E-MTAB-12685 [https://www.ebi.ac.uk/biostudies/arrayexpress/studies/E-MTAB-12685]. All other relevant data supporting the key findings of this study are available
within the article and its Supplementary Information files or from the corresponding author upon reasonable request. A reporting summary for this Article is available as a Supplementary Information file.
Variants between B6 and CAST mouse strains are available at ftp://ftp-mouse.sanger.ac.uk/current_snps/mgp.v5.merged.snps_all.dbSNP142.vcf.gz. Genomic files and annotations are available from ensembl http://www.ensembl.org/Mus_musculus/Info/Index.

Research involving human participants, their data, or biological material

Policy information about studies with human participants or human data. See also policy information about sex, gender (identity/presentation), and sexual orientation and race, ethnicity and racism.

Reporting on sex and gender	This research does not involve human participants, their data or biological material.
Reporting on race, ethnicity, or other socially relevant groupings	This research does not involve human participants, their data or biological material.
Population characteristics	This research does not involve human participants, their data or biological material.
Recruitment	This research does not involve human participants, their data or biological material.
Ethics oversight	This research does not involve human participants, their data or biological material.

Note that full information on the approval of the study protocol must also be provided in the manuscript.

Field-specific reporting

Please select the one below that is the best fit for your research. If you are not sure, read the appropriate sections before making your selection.

☒ Life sciences ☐ Behavioural & social sciences ☐ Ecological, evolutionary & environmental sciences

For a reference copy of the document with all sections, see nature.com/documents/nr-reporting-summary-flat.pdf

Life sciences study design

All studies must disclose on these points even when the disclosure is negative.

Sample size	No sample-size calculations were performed. We use the same number of replicates as in a previous similar study design (Goncalves et al., Genome Research, 2012), which constitutes a high number of biological replicates for single-cell RNA-Sequencing experiments compared to the field. We validate general genome-wide trends with smaller sample sizes (two replicates).
Data exclusions	No exclusion of sequencing datasets was performed. Within each single-cell RNA-Sequencing samples, cells with a low quality of sequencing data (less than 500 detected UMIs or genes) were excluded.
Replication	We distributed the six biological replicates used across three experimental groups and find strong concordance in the obtained datasets. We validate some findings using orthogonal approaches (ATAC-Seq) or a third species (Mus caroli).
Randomization	No experiments were performed where randomization applies, as no treatments or other procedures are involved.
Blinding	No blinding was performed during collection or analysis.

Reporting for specific materials, systems and methods

We require information from authors about some types of materials, experimental systems and methods used in many studies. Here, indicate whether each material, system or method listed is relevant to your study. If you are not sure if a list item applies to your research, read the appropriate section before selecting a response.

Materials & experimental systems

n/a	Involved in the study
☒	☐ Antibodies
☒	☐ Eukaryotic cell lines
☒	☐ Palaeontology and archaeology
☐	☒ Animals and other organisms
☒	☐ Clinical data
☒	☐ Dual use research of concern
☒	☐ Plants

Methods

n/a	Involved in the study
☒	☐ ChIP-seq
☒	☐ Flow cytometry
☒	☐ MRI-based neuroimaging

Animals and other research organisms

Policy information about studies involving animals; ARRIVE guidelines recommended for reporting animal research, and Sex and Gender in Research

Laboratory animals	Mice (Mus musculus domesticus, Mus musculus castaneus and Mus caroli) are of C57BL/6-Ly5.1, CAST/EiJ and CAROLI/EiJ strains, sacrificed after 8 weeks of age. All mice were bred in-house in the animal facilities of DKFZ under specific pathogen-free conditions in individually ventilated cages at 24°, a humidity of 80% with fixed day/night cycles of 12h.
Wild animals	No wild animals were used.
Reporting on sex	The presented study concerns spermatogenesis and therefore intrinsically only applies to male mice.
Field-collected samples	No field collected samples were used.
Ethics oversight	Mice were held in the animal facilities of the DKFZ under specific pathogen-free conditions or in the Biological Resources Unit (BRU) in the Cancer Research UK – Cambridge Institute under Home Office Licence PPL 70/7535. Mice were euthanized by cervical dislocation and all animal procedures were performed according to protocols approved by the Regierungspräsidium Karlsruhe.

Note that full information on the approval of the study protocol must also be provided in the manuscript.

Plants

Seed stocks	Not applicable.
Novel plant genotypes	Not applicable.
Authentication	Not applicable.
